# Transcriptome analysis and mining of genes related to shade tolerance in foxtail millet (*Setaria italica* (L.) P. Beauv.)

**DOI:** 10.1098/rsos.220953

**Published:** 2022-10-05

**Authors:** Dan Liu, Yanjiao Cui, Zilong Zhao, Jing Zhang, Suying Li, Zhengli Liu

**Affiliations:** ^1^ Department of Life Sciences, Tangshan Normal University, Tangshan, People's Republic of China; ^2^ Tianjin Key Laboratory of Crop Genetics and Breeding, Institute of Crop Sciences, Tianjin Academy of Agricultural Sciences, Tianjin, People's Republic of China

**Keywords:** Chinese chestnut, differentially expressed genes, foxtail millet, net photosynthetic rate, shade tolerance, weighted gene co-expression network analysis

## Abstract

A stereo interplanting system with foxtail millet beneath chestnut trees is an effective planting method to raise the utilization of land in chestnut orchards, increase yields and improve quality of chestnut nuts. Consequently, exploration of genes involved in shade tolerance response in foxtail millet and breeding shade-tolerant varieties have become urgent issues. In this study, RNA-seq of leaf samples from two shade-tolerant varieties and three shade-intolerant varieties of foxtail millet at the booting stage was performed. Comparisons between the varieties revealed that 70 genes were commonly differentially expressed. Moreover, the ratio of net photosynthetic rate under shaded environment to that under light environment could be used as an indicator of shade tolerance. Subsequently, weighted gene co-expression network analysis was employed to construct a co-expression network and modules were correlated with this ratio. A total of 375 genes were identified as potentially relevant to shade tolerance, among which nine genes were also present in the 70 differentially expressed genes, which implied that they were good candidates for genes involved in shade tolerance. Our results provide valuable resources for elucidation of the molecular mechanisms underlying shade tolerance and will contribute to breeding of shade-tolerant foxtail millet that are adapted to the shaded environment under chestnut trees.

## 1. Introduction

Chinese chestnut (*Castanea mollissima* Blume), an economically important *Castanea* species with high nutritional value, originated from China and has been cultivated for more than 2500 years [1–3]. The wide distribution of Chinese chestnut in China extends from 41° N in the north, following the ranges of the Yanshan Mountains, to 18° N in the south, following the Wuzhi Mountain of Hainan Island [4]. Among these areas, land resources are scarce, while the large amounts of land under canopies of chestnut trees are always deserted and overgrown with weeds [5,6]. In addition, chestnut trees are often severely attacked by pests and diseases, leading to significant reductions in output and quality of chestnut nuts [7,8]. A stereo interplanting system is an optimized and efficient planting pattern, which can realize multi-layered and comprehensive utilization in space, time and function of a variety of coordinated and interconnected agricultural living things, such as high-stem and dwarf plants, sun-loving and shade-tolerant plants. Therefore, it may offer a convenient strategy for efficiently using the land under the spreading chestnut tree, increasing yields and economic returns, and improving the quality of chestnut nuts and crops under the trees.

According to our field survey, several crops, such as maize (*Zea mays* L.), sorghum (*Sorghum bicolor* (L.) Moench), soya bean (*Glycine max* L.) and peanut (*Arachis hypogaea* L.), have been used by farmers for interplanting with chestnut in recent years. However, these crops usually did not grow well in the shaded conditions under the trees and consequently yields of the crops are low. Moreover, the growth period of these crops is too long to achieve the coupling of critical developmental stages with that of chestnut, and this then affects the harvest of chestnut nuts. Identifying ideal species for interplanting beneath chestnut trees has thus become an urgent problem.

Foxtail millet (*Setaria italica* (L.) P. Beauv.) is a traditional cereal food crop in China that has wide adaptability and can be sown in spring, late spring and summer [9]. The growth period of foxtail millet sown in spring almost overlaps with that of chestnut. Moreover, this crop requires minimum water and fertilizer input for growth, has strong tolerance to drought and barren soil, and has significant economic benefits [9]. Therefore, foxtail millet has obvious advantages when compared with other crops and is, therefore, a potential candidate for the stereo interplanting system of chestnut. In previous work, we bred several foxtail millet materials and found that only early maturing, shade-tolerant materials, which also had a high content of crude protein, low content of tannin and thick wax layer on their leaves, could be used for interplanting with chestnut in practice. When interplanted with chestnut, such foxtail millet materials could attract red spiders to feed off the foxtail millet, which led to a 5–15% decrease in the rate of bad chestnut nuts caused by red spiders, while the damage caused by red spiders to foxtail millet is small [10]. This stereo interplanting method makes full use of the land resources under canopies of chestnut trees and improves the quality of chestnut nuts, as well as expanding the space for development of foxtail millet as the planting area of this crop has been shrinking gradually in recent years. The method is also popular with farmers. Consequently, cultivation of new varieties and hybrids of foxtail millet adapted to the shaded environment under chestnut trees is a key factor restricting the development of the stereo interplanting system with foxtail millet beneath chestnut trees. Screening and identification of genes related to shade tolerance in foxtail millet will facilitate the development of such novel varieties and hybrids to overcome this problem.

Shade tolerance is a concept that refers to the capacity of a plant to grow in shade, and is measured by the average lowest light levels at which a particular species can survive in dense vegetation [11–13]. In dense vegetation, there is a drop in the red (R, 660 nm) to far-red (FR, 730 nm) ratio (R : FR) and this triggers one of two different strategies to cope with shade [14]. In many plants that are of similar heights, such as in grasslands, the change in R : FR ratio induces a suite of traits termed shade avoidance syndrome (SAS). This strategy includes elongation of stem-like organs (hypocotyls and petioles), apical dominance, upward movement of leaves (hyponasty) and reduced branching [15,16]. By contrast, species that thrive on the forest floor and cannot outgrow the tall trees surrounding them, such as in forest understories, use the shade tolerance response (STR), adapting their phenotype to cope with shaded environments [17,18]. This strategy consists of increased specific leaf area (SLA), photosystem (PS) II : PSI ratio, physical defence, and reduced chlorophyll a : b ratio [17].

Unlike SAS, STR and its molecular regulation remain poorly understood. Comparative RNA sequencing of two *Geranium* species, *G*. *pyrenaicum* (hedge cranesbill) and *G. robertianum* (herb Robert), that respond to low R : FR light conditions with contrasting ecological strategies revealed that STR shares a common phytochrome-mediated mechanism with SAS. Upon shade detection, the genes controlling plant immunity and shoot elongation, which act downstream of the phytochrome gene *PhyB*, exhibited contrasting patterns of expression [18]. Furthermore, several genes required for hormone signalling pathways were reported to be involved in STR regulation. For example, Li *et al*. [19] treated a shade-tolerant mutant of perennial ryegrass (*Lolium perenne* L.; *shadow-1*) and wild-type plants with 95% shade for two weeks, and compared their transcriptomes with those exposed to full light. The results showed that expression of gibberellin (GA) biosynthesis genes and one GA response gene was downregulated in *shadow-1* plants under shade conditions when compared with wild-type, suggesting a possible role of GA in shade tolerance. Recently, the rate of panicle emergence of shade-tolerant Swarnaprabha (SP) rice in shade was observed to be higher than that of control plants, and most of the genes involved in ethylene and cytokinin signalling pathways were upregulated in the shade-grown panicles of SP [20]. This indicated a positive role for these genes in STR in SP. However, the genes involved in STR in foxtail millet and the molecular underpinnings have yet to be elucidated.

To explore the genes involved in STR in foxtail millet, the transcriptomes of two shade-tolerant varieties and three shade-intolerant varieties of this cereal crop were generated and compared in the present study. Through examination of differential gene expression and construction of gene co-expression modules, several candidate genes that potentially contribute to STR in foxtail millet were identified and gene ontology (GO) and Kyoto Encyclopaedia of Genes and Genomes (KEGG) enrichment analyses were performed on these genes. Findings from the study provide new insights towards elucidation of the biological pathways and molecular mechanisms underlying STR in foxtail millet and highlight possible strategies for developing shade-tolerant plants.

## 2. Results

### 2.1. Experimental design and overview of RNA sequencing data

To screen for foxtail millet varieties with different levels of shade tolerance, the yields of 22 varieties grown in an open field or in shaded conditions under chestnut trees of 10 and 15 years old were determined (electronic supplementary material, table S1). Based on the findings from our survey, varieties with yields that were stable between light and shaded environments were defined as shade-tolerant varieties, namely whose yield change under 10-year-old chestnut trees compared with in an open field was less than 5%, and the yield change under 15-year-old chestnut trees was less than 7%, otherwise were defined as shade-intolerant varieties. Among the 22 varieties, two shade-tolerant varieties, Tangzagu 56229 and Tangzagu 51950, and three shade-intolerant varieties, Tangzagu 12950, Tangzagu 57295 and Tangzagu 1121, were selected.

To identify genes involved in STR of foxtail millet, 15 cDNA libraries were constructed from the first, second and third leaf samples from the top of these five varieties at booting stage, which were grown in an open field, and each sample was measured in three biological replicates. The libraries were sequenced using an Illumina Novaseq PE150 platform and a total of 645.17 M raw reads were generated from the 15 samples. After discarding adaptor sequences, duplicated reads and low-quality reads, 641.29 M clean reads were obtained, and the percentage of clean reads in each library ranged from 96.97% to 99.76% (table 1). The clean reads were aligned to the foxtail millet reference genome using HISAT2 v. 2.0.6 [21], and approximately 94.76–95.75% of the clean reads mapped to the reference genome [22] (table 1). According to Schuierer *et al*. [23], the high mapping rate suggested that the quality of RNA-seq was good and the results were reliable. Moreover, the Pearson's correlation coefficients between biological replicates were calculated. As shown in electronic supplementary material, table S2, the biological replicates were highly correlated, with all correlations over 0.996. These results further indicated that there was sufficient reproducibility and robustness of the biological replicates and RNA-seq results, which made them suitable for the following analysis.

**Table 1.** Summary of RNA-seq data for leaf samples of five foxtail millet varieties at booting stage. The suffixes -1, -2, -3 indicate three biological replicates for each sample.

sample	raw reads (M)	clean reads (M)	per cent (%)	clean bases (G)	mapped reads (%)
Tangzagu 56229-1	37.857	37.768	99.76	11.257	95.75
Tangzagu 56229-2	43.064	42.930	99.69	12.841	95.49
Tangzagu 56229-3	44.932	44.766	99.63	13.378	95.30
Tangzagu 51950-1	41.169	40.978	99.54	12.250	95.41
Tangzagu 51950-2	41.962	41.832	99.69	12.514	95.33
Tangzagu 51950-3	43.031	42.884	99.66	12.837	95.38
Tangzagu 12950-1	38.042	37.692	99.08	11.261	94.76
Tangzagu 12950-2	47.110	46.880	99.51	13.999	95.28
Tangzagu 12950-3	44.196	43.962	99.47	13.134	95.27
Tangzagu 57295-1	41.444	40.187	96.97	12.023	95.39
Tangzagu 57295-2	39.790	39.688	99.74	11.865	95.48
Tangzagu 57295-3	52.574	52.112	99.12	15.523	95.65
Tangzagu 1121-1	41.028	40.871	99.62	12.224	94.85
Tangzagu 1121-2	50.023	49.902	99.76	14.918	94.94
Tangzagu 1121-3	38.949	38.839	99.72	11.622	94.93

**Figure 1.**
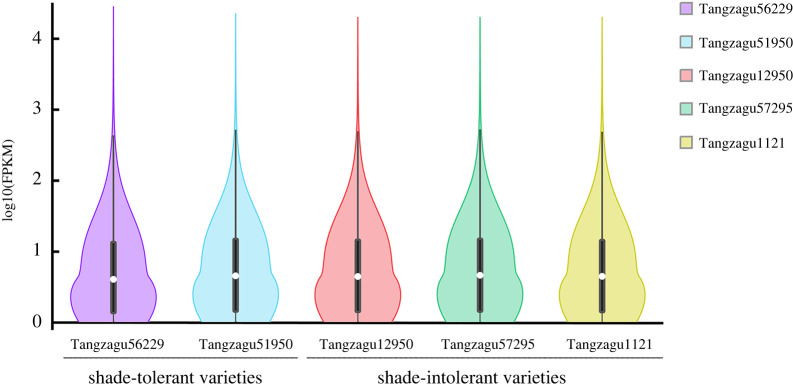
Violin plot of gene expression levels in two shade-tolerant and three shade-intolerant varieties of foxtail millet. FPKM, fragments per kilobase of transcript per million fragments mapped.

A total of 30 344 genes were obtained following assembly of the mapped sequences in each library, and the expression level of each gene was estimated using fragments per kilobase of transcript per million fragments mapped (FPKM) [24]. Gene expression levels were highest in Tangzagu 12950 (median: 1.28 FPKM), followed by Tangzagu 51950 (median: 1.22 FPKM), Tangzagu 1121 (median: 1.13 FPKM) and Tangzagu 57295 (median: 1.12 FPKM), with the lowest expression levels detected in Tangzagu 56229 (median: 0.93 FPKM) (figure 1).

### 2.2. Differential gene expression

**Figure 2.**
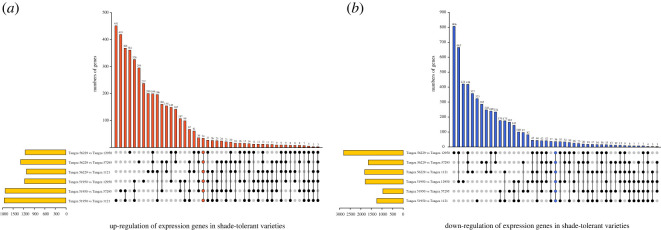
Diagram depicting the number of differentially expressed genes (DEGs) between shade-tolerant and shade-intolerant varieties of foxtail millet. The distribution of upregulated (*a*) and downregulated (*b*) DEGs in each comparison is shown.

**Figure 3.**
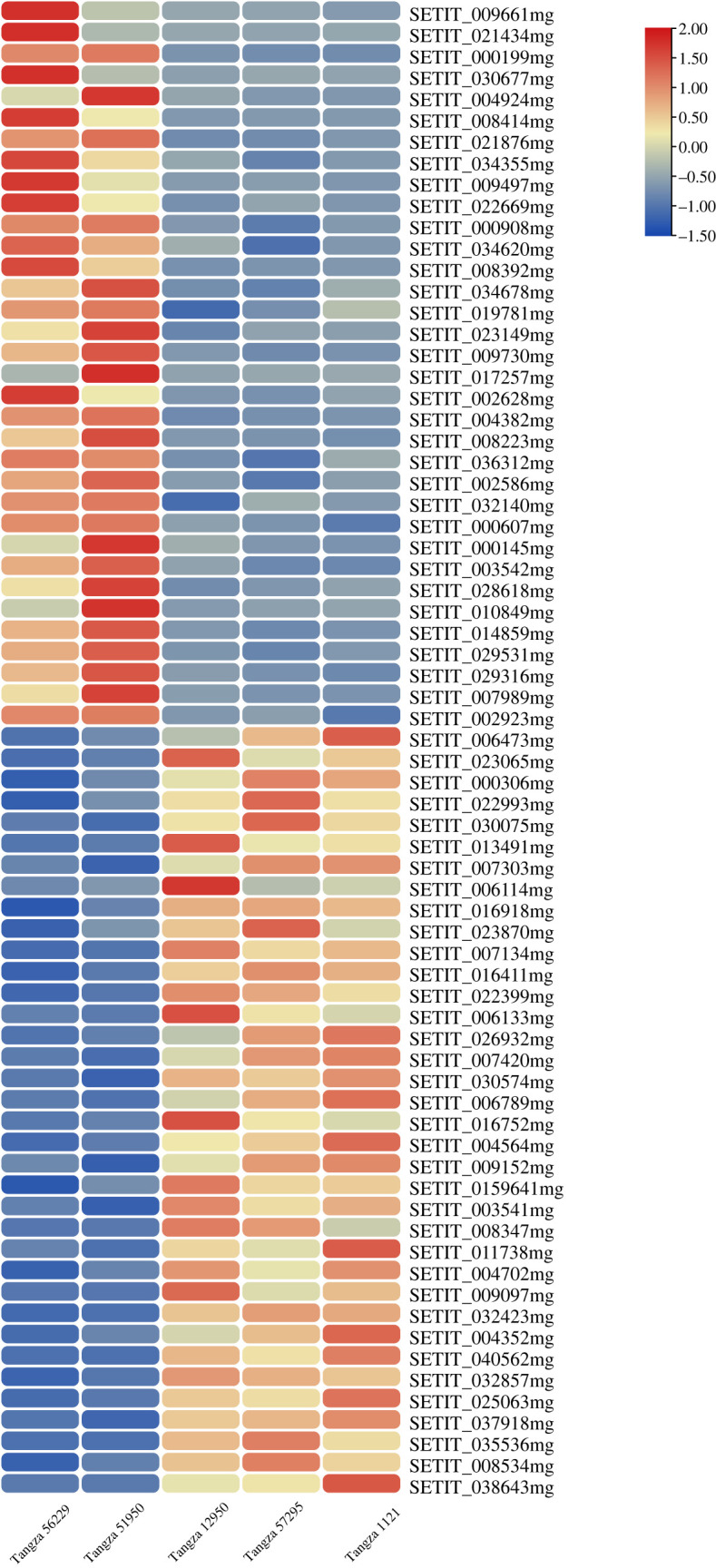
Cluster analysis by FPKM value of the differentially expressed genes commonly existing in each comparison. Red indicates higher expression and blue indicates lower expression.

To explore the genes involved in STR of foxtail millet, differentially expressed genes (DEGs) between shade-tolerant varieties (Tangzagu 56229 and Tangzagu 51950) and shade-intolerant varieties (Tangzagu 12950, Tangzagu 57295 and Tangzagu 1121) were detected using edgeR v. 3.3.3 [25], with the genes that showed a |log_2_(fold change)| > 1 and *p*adj < 0.05 being defined as DEGs. As shown in figure 2, there were 3971, 2936 and 2943 DEGs in shade-tolerant variety Tangzagu 56229 when compared with Tangzagu 12950, Tangzagu 57295 and Tangzagu 1121, respectively, with 1158, 1289 and 1124 genes upregulated, respectively, and 2813, 1647 and 1819 genes downregulated, respectively. Similarly, 2964, 2688 and 3004 DEGs were found in shade-tolerant variety Tangzagu 51950 when compared with Tangzagu 12950, Tangzagu 57295 and Tangzagu 1121, respectively, with 1173, 1726 and 1754 genes presenting an upregulated pattern, respectively, and 1791, 962 and 1250 genes presenting a downregulated pattern, respectively (figure 2). Seventy DEGs were shared in each comparison (34 upregulated and 36 downregulated), and heatmap clustering analysis for these DEGs was performed (figure 3).

### 2.3. Chromosome location of differentially expressed genes

**Figure 4.**
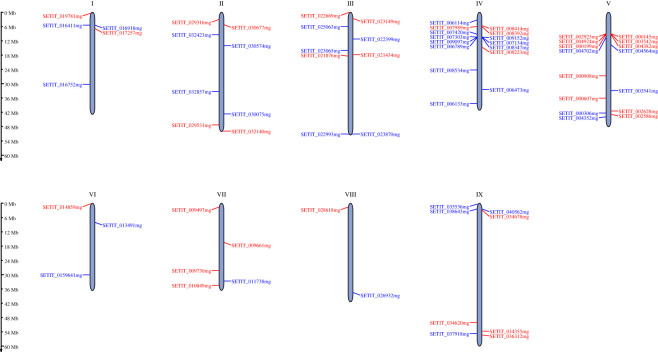
Chromosomal distribution of the differentially expressed genes (DEGs) commonly existing in each comparison. The chromosome number is indicated at the top of each chromosome, and the scale bar indicates chromosome length. Red represents upregulated DEGs in shade-tolerant varieties, and blue represents downregulated DEGs.

Distributions of the 70 shared DEGs were detected and the genes were found to map diffusely on nine chromosomes of foxtail millet (figure 4). The number of DEGs on each chromosome ranged from 2 to 15. Chromosomes IV and V possessed the largest number of DEGs, while chromosome VIII only contained two DEGs. Most DEGs were located on the proximal or distal ends of foxtail millet chromosomes. Several DEGs located on the same chromosome in clusters, such as *SETIT_002923mg*, *SETIT_004924mg*, *SETIT_000199mg* and *SETIT_004702mg* on chromosome V.

### 2.4. Quantitative real-time PCR verification

**Figure 5.**
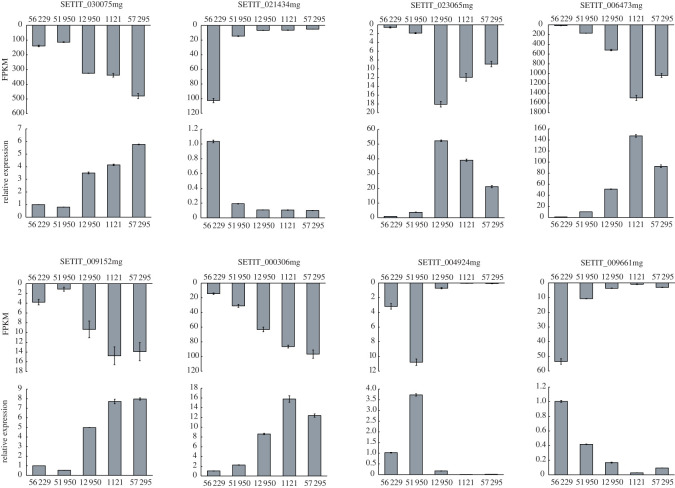
Verification of eight differentially expressed genes (DEGs) by qRT-PCR. The *X*-axis represents different foxtail millet varieties, and the *Y*-axis indicates the expression level of genes in RNA-seq data (evaluated by FPKM) and relative expression level of selected genes determined by qRT-PCR. The *SiACTIN* gene was used as an internal control, and the transcript level of genes in variety 56229 was set as 1.0. Error bars represent standard error (*n* = 3).

To verify the reliability of gene changes in transcriptome analysis, quantitative real-time PCR (qRT-PCR) technology was applied to analyse the transcriptional levels of eight randomly selected DEGs, including three genes which were predicted upregulated in shade-tolerant varieties (*SETIT_021434mg*, *SETIT_004924mg*, *SETIT_009661mg*), and five genes which were predicted downregulated (*SETIT_030075mg*, *SETIT_023065mg*, *SETIT_006473mg*, *SETIT_009152mg*, *SETIT_000306mg*). As shown in figure 5, the alteration pattern of these genes was consistent with that of transcriptome analysis, indicating that our RNA-seq data is reliable.

### 2.5. Gene ontology and Kyoto Encyclopaedia of Genes and Genomes enrichment analysis of DEGs

**Figure 6.**
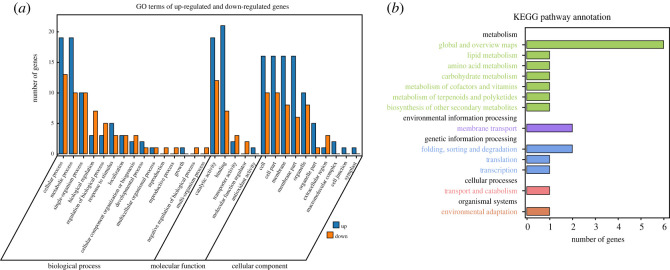
GO and KEGG enrichment analyses of differentially expressed genes (DEGs) commonly existing in each comparison. (*a*) Enriched GO terms of biological processes, molecular function and cellular components are shown. Blue indicates upregulated DEGs in shade-tolerant varieties, and red indicates downregulated DEGs. (*b*) Enriched KEGG pathways. The numbers of DEGs are shown in the *X*-axis, and the KEGG pathway terms are shown in the *Y*-axis.

The distribution of 70 DEGs in GO terms was examined by analysis of the enrichment gene set (electronic supplementary material, table S3) to identify possible functions of the genes in foxtail millet. The 70 DEGs could be annotated to 30 GO terms (figure 6*a*). Based on the biological process term frequencies, the top three terms were cellular process (GO:0009987), metabolic process (GO:0008152) and single-organism process (GO:0044699), which may indicate that these DEGs function in multiple biological processes. In the molecular function section, the majority of DEGs were enriched to catalytic activity (GO:0003824) and binding (GO:0005488), suggesting that genes assigned to these two terms required binding or interaction to perform their structural or functional activities. In the cellular component section, the top five terms were cell (GO:0005623), cell part (GO:0044464), membrane (GO:0016020), membrane part (GO:0044425) and organelle (GO:0043226). This implied that some proteins located on cell membranes and membrane-enclosed cellular organelles, such as mitochondria and chloroplasts, may greatly contribute to shade tolerance in foxtail millet.

To further understand potential regulatory roles of the DEGs in shade tolerance of foxtail millet, KEGG pathway analyses were performed. A total of 13 pathways were enriched, among which seven (53.85%), three (23.08%), one (7.69%), one (7.69%) and one (7.69%) were related to metabolism, genetic information processing, environmental information processing, cellular processes and organismal systems, respectively (figure 6*b*). Several DEGs were found to be associated with pathways related to starch and sucrose metabolism (ko00500) and carotenoid biosynthesis (ko00906) (electronic supplementary material, table S4), which referred to important products and pigment involved in photosynthesis, suggesting a possible effect of photosynthesis in STR of foxtail millet.

### 2.6. Determination of net photosynthetic rate

To verify the association of STR of foxtail millet with photosynthetic characteristics, the net photosynthetic rates of the 22 varieties used for yield trait evaluation were measured. The net photosynthetic rates of shade-tolerant varieties (Tangzagu 56229 and Tangzagu 51950) decreased less in shaded environments (33.74% and 29.82% under 10-year-old chestnut trees, respectively, and 72.66% and 73.33% under 15-year-old chestnut trees, respectively) and exhibited more stable photosynthetic characteristics, while the net photosynthetic rates of shade-intolerant varieties (Tangzagu 12950, Tangzagu 57295 and Tangzagu 1121) decreased 67.18%, 59.46% and 67.28% under 10-year-old chestnut trees and 79.41%, 79.83% and 81.85% under 15-year-old chestnut trees, respectively (table 2). These results revealed that stronger shade tolerance was accompanied by a more stable net photosynthetic rate in foxtail millet, and the change in net photosynthetic rate under shaded conditions may be used as an indicator of shade tolerance in foxtail millet.

**Table 2.** Net photosynthetic rates of 22 foxtail millet varieties used in this study. The ratio was defined as the net photosynthetic rate under shaded environment divided by that in open field.

variety	in open field	in shaded conditions under 10-year-old chestnut trees		in shaded conditions under 15-year-old chestnut trees	
	net photosynthetic rate (μmol m^−2^ s^−1^)	net photosynthetic rate (μmol m^−2^ s^−1^)	ratio	net photosynthetic rate (μmol m^−2^ s^−1^)	ratio
HK950	31.45	11.46	0.37	6.90	0.22
Tangzagu 5664	33.33	14.58	0.45	5.56	0.17
Tangzagu 2011	29.91	13.60	0.47	7.35	0.25
Tangzagu 3739	27.16	13.81	0.52	7.04	0.26
Tangzagu 56229	23.54	15.60	0.68	6.44	0.28
Tangzagu 3495	28.52	18.05	0.65	5.95	0.22
Tangzagu 51950	24.14	16.94	0.70	6.44	0.27
JG31	25.85	12.10	0.48	6.29	0.25
Tangzagu 56902	20.60	12.64	0.62	6.40	0.31
Tangzagu 12950	33.40	10.96	0.33	6.88	0.21
Tangzagu 3227	32.83	11.15	0.35	7.98	0.25
Tangzagu 5695	31.25	13.48	0.44	6.95	0.22
Tangzagu 5168	28.40	13.96	0.50	7.05	0.25
Tangzagu 57295	33.40	13.54	0.41	6.74	0.20
Tangzagu 1121	36.49	11.94	0.33	6.62	0.18
Tangzagu 386950	28.86	11.77	0.41	7.24	0.25
Tangzagu 386902	28.40	11.57	0.41	7.17	0.25
Tangzagu 899902	26.16	11.07	0.42	5.93	0.23
Tangzagu 899808	24.97	10.63	0.43	7.27	0.30
Tangzagu 201808	26.10	9.78	0.38	6.14	0.24
Tangzagu 40892	26.05	8.93	0.35	5.56	0.22
Tangzagu 201892	23.18	8.53	0.38	5.18	0.23

### 2.7. Construction of gene co-expression network and identification of key modules

**Figure 7.**
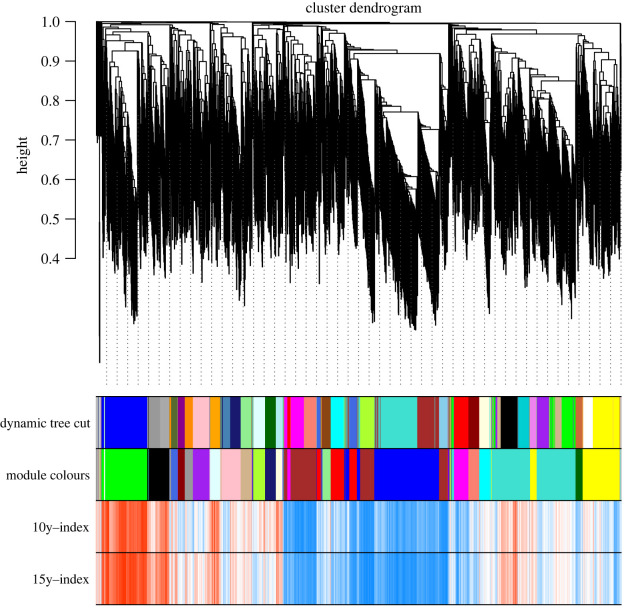
Clustering dendrogram of genes, with dissimilarity based on topological overlap, together with assigned module colours.

**Figure 8.**
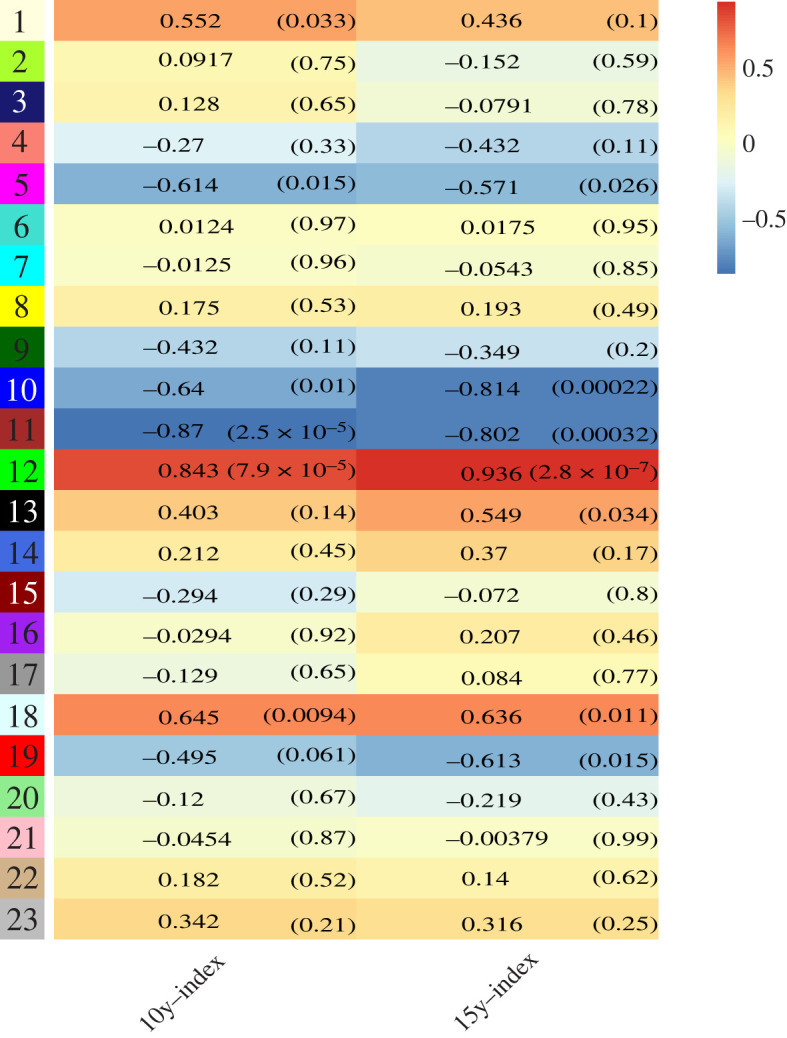
Module–trait associations. Each row corresponds to a module, and the two columns correspond to the ratio of net photosynthetic rate of foxtail millet grown in shaded environment (under chestnut trees of 10 and 15 years old) to that in light environment. Each cell contains the corresponding correlation and *p-*value. The table is colour-coded by correlation according to the colour legend. Intensity and direction of correlations are indicated on the right side of the heatmap (red, positively correlated; blue, negatively correlated).

Genes exhibiting similar expression patterns may be involved in similar biological processes or networks [26]. To understand the gene expression network during STR of foxtail millet, the expression values of expressed genes (FPKM > 1) identified in this study were used to construct co-expression networks. An appropriate soft thresholding value can effectively reduce the correlation noise, making the network conform to scale-free network attributes [27]. In the current study, optimal soft thresholding value of *β* was 28 with *R*^2^ as 0.85 (electronic supplementary material, figure S1). As shown in figure 7, 23 gene co-expression modules were identified using weighted gene co-expression network analysis (WGCNA), and each module contained a set of genes showing significant expression correlations with each other. The modules were then correlated with the ratio of net photosynthetic rate under shaded environment to that under light environment, which reflected the shade tolerance, and the most significant associations were identified. The module 12 with 169 genes was most significantly and positively correlated with shade tolerance, while the module 11 with 206 genes was most significantly and negatively correlated with shade tolerance (figure 8).

### 2.8. Functional analysis of key modules

**Figure 9.**
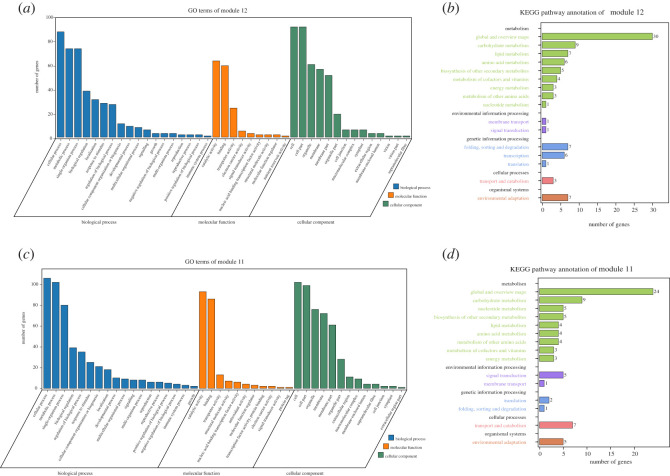
GO and KEGG enrichment analyses of genes involved in module 12 and module 11. Enriched GO terms of biological processes, molecular function and cellular components are shown in (*a*) and (*c*); enriched KEGG pathways are shown in (*b*) and (*d*). Number of genes are shown in the *X*-axis, and the KEGG pathway terms are shown in the *Y*-axis.

GO analysis of the 169 genes in module 12 and 206 genes in module 11 was conducted (figure 9*a*,*c*, electronic supplementary material, tables S5 and S6). The analysis revealed that for biological process, the top three terms were cellular process (GO: 0009987), metabolic process (GO:0008152) and single-organism process (GO:0044699); for molecular function, the genes were mainly enriched in catalytic activity (GO:0003824) and binding (GO:0005488); and for cellular component, the top five terms were cell (GO:0005623), cell part (GO:0044464), organelle (GO:0043226), membrane (GO:0016020) and membrane part (GO:0044425). These results were consistent with the GO analysis of the 70 DEGs (figure 6*a*).

KEGG analysis of the genes in module 12 was then performed and 16 module-regulated pathways were identified, including nine (56.25%) related to metabolism, two (12.50%) related to environmental information processing, three (18.75%) related to genetic information processing and one (6.25%) related to each of cellular processes and organismal systems, respectively (figure 9*b*, electronic supplementary material, table S7). Similarly, 15 module-regulated pathways were identified in module 11, which comprised nine pathways (60%) related to metabolism, two (13.33%) related to environmental information processing and genetic information processing, respectively, and one (6.67%) related to cellular processes and organismal systems, respectively (figure 9*d*, electronic supplementary material, table S8).

### 2.9. Identification of key genes involved in shade tolerance response

**Figure 10.**
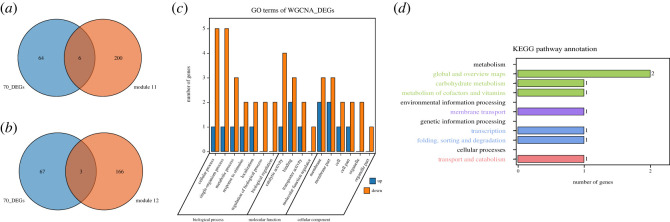
Identification and functional enrichment analysis of key genes that participate in regulation of shade tolerance in foxtail millet. (*a*) and (*b*) Venn diagrams showing the overlapping genes between the differentially expressed genes commonly existing in each comparison and genes in module 11 and module 12, respectively (blue, upregulated genes; red, downregulated genes). (*c*) GO enrichment analysis of potential key genes involved in shade tolerance regulation. (*d*) KEGG enrichment analysis of potential key genes involved in shade tolerance regulation. Number of genes are shown in the *X*-axis, and the KEGG pathway terms are shown in the *Y*-axis.

To determine the key genes related to STR in foxtail millet, the 70 DEGs were compared with the 169 genes of module 12 and 206 genes of module 11. Nine genes were found to be shared between them and were considered as key candidates in STR (figure 10*a*,*b*). Cluster analysis of DEGs by FPKM values showed that among the nine genes, three were upregulated and six were downregulated in shade-tolerant varieties of foxtail millet (figure 3).

The regulatory role of these nine shared genes was explored using GO and KEGG enrichment analyses (figure 10*c*,*d*, electronic supplementary material, tables S9 and S10). In the GO analysis, for the biological process section, the genes were annotated to cellular process (GO:0009987), single-organism process (GO:0044699), metabolic process (GO:0008152), response to stimulus (GO:0050896), localization (GO:0051179), regulation of biological process (GO:0050789) and biological regulation (GO:0065007). Regarding molecular function, the genes were enriched in catalytic activity (GO:0003824), binding (GO:0005488), transporter activity (GO:0005215) and molecular function regulator (GO:0098772), while for cellular component, the genes were distributed in membrane (GO:0016020), membrane part (GO:0044425), cell (GO:0005623), cell part (GO:0044464), organelle (GO:0043226) and organelle part (GO:0044422). The KEGG analysis identified seven pathways, including three (42.85%) related to metabolism, one (14.29%) related to environmental information processing, two (28.57%) related to genetic information processing and one (14.29%) related to cellular processes (figure 10*d*).

## 3. Discussion

In our previous work, several shade-tolerant varieties of foxtail millet were bred and all had a high content of crude protein, a low content of tannin and a thick wax layer on their leaves. These characteristics could attract red spiders to feed; red spider is one of the predominant pests of Chinese chestnut [8,28,29], thus these foxtail millet varieties reduced the damage to chestnut trees and improved the quality of chestnut nuts. Therefore, shade-tolerant foxtail millet varieties are an attractive candidate for interplanting with Chinese chestnut. The identification and cloning of genes related to shade tolerance and the subsequent breeding of shade-tolerant varieties that can grow well in the shaded environment under chestnut trees has consequently attracted considerable attention. In this study, the transcriptomes of two shade-tolerant foxtail millet varieties and three shade-intolerant varieties kept under light were analysed. Through Illumina sequencing, millions of clean reads were generated, which represented gigabytes of sequencing data. These reads were efficiently mapped to the foxtail millet reference genome, and the biological replicates were highly correlated, demonstrating the robustness and reliability of the data (table 1, electronic supplementary material, table S2). The transcriptome data acquired from shade-tolerant and shade-intolerant varieties of foxtail millet under light conditions provide valuable resources for exploring potential candidate genes and genetic mechanisms underlying STR in foxtail millet and are useful to breeders who are interested in developing new shade-tolerant varieties.

To identify the potential candidate genes involved in STR of foxtail millet, expression profiling between shade-tolerant and shade-intolerant varieties were compared and revealed 70 shared DEGs in each comparison, including a gene encoding heat shock 70 kDa protein 8 (*HSP70-8*, *SETIT_034355mg*), which belongs to the 70 kDa heat-shock protein (HSP70) family. HSP70 proteins are highly conserved molecular chaperones with diverse functions, including widespread involvement in abiotic stress and hormone responses in different species [30–32]. For example, a putative heat-shock transcription factor (HSF) in *Arabidopsis*, *HsfA2*, was reported to be induced by high-light and heat-shock stress. The putative *HSP70* gene (*At3g12580*) served as a target gene of *HsfA2* and was upregulated in *HsfA2*-overexpressing plants, suggesting a possible role of *HSP70* in light stress response [33]. Furthermore, several *HSP70* genes in foxtail millet were also reported to be differentially expressed in response to abiotic stresses, such as dehydration, heat, salinity and cold, implying their potential role in environmental adaptations [34,35]. Recently, An *et al*. [36] identified that eight *HSP* genes were differentially expressed in *Phoebe bournei* due to low light intensity at 35% and 10% light exposure through transcriptome analysis, among which most *HSP70* genes were downregulated under low light stress compared with CK (100% light intensity), contributing to shade tolerance by inhibiting unnecessary defence mechanism. Combined with its differential expression in shade-tolerant varieties, *HSP70-8* in foxtail millet may function in STR, which is induced by a low R : FR ratio in shaded environments. However, further studies are needed to verify our hypothesis and elucidate the mechanism.

WGCNA is an effective method to explore the relationships between genes and phenotypes, through which genes are clustered into co-expressed modules and a bridge between sample characteristics and changes in gene expression can be established [37]. It has been widely applied to discover candidate hub genes related to target traits in plant species, such as co-expressed genes responsive to abiotic and biotic stress in *Arabidopsis*, rice and maize [38–40], genes associated with spike complexity and yield in wheat [41,42] and genes related to anthocyanin biosynthesis in eggplant [43]. As the results of the current study supported that the change of net photosynthetic rate under shaded conditions may provide an indication of shade tolerance (table 2), the expression values of expressed genes identified in the study were used to build a co-expression network by WGCNA, and modules were correlated with the ratio of net photosynthetic rate under shaded environment to that in light environment to filter the key genes. A total of 375 genes were identified as potentially relevant to STR by WGCNA, including a gene encoding aminopropyl transferase (*SETIT_006576mg*). Spermidine synthase (SPDS) and spermine synthase (SPMS) belong to the aminopropyl transferases, a class of widely distributed enzymes that use decarboxylated S-adenosylmethionine (dcSAM) and putrescine (Put) or spermidine (Spd) as an aminopropyl donor and an amino acceptor, respectively, to form Spd or spermine (Spm) in that order [44]. Several studies revealed that these aminopropyl transferase genes in plants are of central relevance to multiple environmental stress tolerance [45–48]. For example, overexpression of the apple *MdSPDS1* gene conferred enhanced tolerance to various stressful conditions, such as salt, osmosis and heavy metal stresses, in transgenic European pear (*Pyrus communis* L.) plants [49]. Moreover, the promoters of *SPDS1* and *SPMS* were reported to contain several ABA-responsive elements [50], and *SPDS1* and *SPMS* genes were strongly upregulated by drought stress [51], suggesting the involvement of ABA in the regulation of *SPDS1* and *SPMS* expression during exposure to drought. Thus, the aminopropyl transferase gene in foxtail millet, *SETIT_006576mg*, may also interact with hormonal pathways and respond to environmental challenges, such as the low R : FR ratio in shaded environments.

Recent studies revealed that accelerated expression of genes related to photosynthesis in *P. bournei* governs the shade tolerance and enables plants to maintain a high photosynthesis rate even under low light conditions [36]. In this study, several genes associated with photosynthesis were detected according to WGCNA analysis, such as *SETIT_024648mg* enriched into light harvesting in photosystem I (GO:000976), *SETIT_036796mg* annotated to photosynthetic electron transport in photosystem I (GO:0009773) and photosystem II assembly (GO:0010207), *SETIT_010591mg* categorized to cytochrome b6f complex assembly (GO:0010190) and photosystem II assembly (GO:0010207) in module 11; and *SETIT_005974mg* annotated to photosystem II repair (GO: 0010206) in module 12. These genes may increase the efficiency of light-trapping molecules and photosynthetic electron transport system in the leaves of shade-tolerant foxtail millet varieties, thereby maintaining more stable effect of photosynthesis (table 2) and yield (electronic supplementary material, table S1) under shaded conditions.

It is worth noting that a β-carbonic anhydrase (CA) gene, *SETIT_002140mg*, was also identified in module 12, which catalyses the reversible interconversion of CO_2_ to HCO_3_^−^, the substrate used by phospho*enol*pyruvate carboxylase (PEPC) in the first step of C_4_ photosynthesis [52]. The reduction of CA accumulation and CA activity results in changes in the abundance of other primary C_4_ pathway enzymes, such as PEPC protein accumulation is significantly increased, malate dehydrogenase and malic enzyme accumulation are decreased, thereby restricting the supply of HCO_3_^−^ to PEPC, and limiting C_4_ photosynthesis and growth [53]. It could be inferred that in the C_4_ crop foxtail millet, *SETIT_002140mg* may be a hub gene which promotes the C_4_ pathway and plant growth by regulating its protein accumulation and activity under shaded environment.

To screen the key genes associated with STR in foxtail millet, the 70 DEGs and 375 genes identified by WGCNA were finally intersected and nine common genes were obtained (figure 9), including the gene *SETIT_006473mg*, which was downregulated in shade-tolerant varieties validated by qRT-PCR (figure 5) and annotated to thiamine biosynthetic process (GO:0009228). The level of thiamine and transcription levels of genes related to thiamine biosynthesis enzymes are enhanced under biotic and abiotic stresses [54–56], suggesting its potential role during STR in foxtail millet, and genetic manipulation of this gene may be an effective strategy for conferring shade tolerance.

## 4. Material and methods

### 4.1. Plant materials

The study employed 22 varieties of foxtail millet. Materials for yield trait evaluation were planted in Beiguan Village and Santun Village of Qianxi County, Tangshan, China in 2020 and 2021. Materials for the measurement of net photosynthetic rate and high-throughput sequencing were planted in Beiguan Village. Yield performance of the 22 varieties of foxtail millet is listed in electronic supplementary material, table S1. Based on their yield change under 10-year-old and 15-year-old chestnut trees compared with in an open field, two shade-tolerant (Tangzagu 56229 and Tangzagu 51950) and three shade-intolerant varieties (Tangzagu 12950, Tangzagu 57295 and Tangzagu 1121) were selected for further high-throughput sequencing.

### 4.2. Whole transcriptome library construction and high-throughput sequencing

The booting stage of foxtail millet is the formation stage of organs related to the economic traits of this plant, and photosynthetic efficiency is highest at this stage. Leaf photosynthetic capacity at this stage directly affects the economic traits of foxtail millet and is closely related to shade tolerance [57]. Therefore, to mine genes related to shade tolerance, leaves at booting stage were sampled from two shade-tolerant varieties of foxtail millet (Tangzagu 56229 and Tangzagu 51950) and three shade-intolerant varieties (Tangzagu 12950, Tangzagu 57295 and Tangzagu 1121). Because chestnut trees grow mainly in mountainous areas, the foxtail millet plants grown in shaded conditions under chestnut trees are susceptible to the influence of microclimate, such as drought stress results from scarce water resources. Therefore, it is challenging to collect samples under shaded conditions and the plants for sampling in this study were all grown in an open field. The middle part of the first, second and third leaves from the top of plants at booting stage were sampled from three standard plants for each variety, and then the collected leaves for each variety were mixed and flash frozen for total RNA isolation. Three biological replicates were performed for each sample.

Total RNA isolation, preparation of whole transcriptome libraries and deep sequencing were performed by Berry Genomics (Beijing, China). Transcriptome libraries were constructed using VAHTS mRNA-seq v. 2 Library Prep Kit for Illumina according to the manufacturer's instructions. The libraries were sequenced on an Illumina NovaSeq platform that generated paired end reads (raw data) of 150 bp.

### 4.3. Transcriptome assembly

The raw data (raw reads) of fastq format were firstly processed through primary quality control using fastp (https://github.com/OpenGene/fastp) [58]. In this step, clean data (clean reads) were obtained by removing read pairs that contain N more than 3 or the proportion of base with quality value below 5 is more than 20%, in any end, or adapter sequence was founded. All the downstream analyses were based on the clean data with high quality. Efficient alignment between the clean reads and the foxtail millet reference genome (Setaria_italica_v2.0, http://plants.ensembl.org/Setaria_italica/Info/Index) [22] was performed using HISAT2 v. 2.0.6 [21], and the mapped reads were then assembled by using Cufflinks v. 2.2.1 [24].

### 4.4. Differential expression analysis

The software edgeR v. 3.3.3 [25] was used to investigate the differential expression of genes between shade-tolerant and shade-intolerant varieties of foxtail millet. Differentially expressed genes (DEGs) were selected with a |log_2_(fold change)| > 1 and adjusted *p*-value (*p*adj) < 0.05. Hierarchical clustering was performed using Cluster 3.0 to generate an overview of the expression profile characteristics. Heatmaps were completed in R (1.20.0) language and environment.

### 4.5. Chromosome location and gene duplication

Chromosome positional data of DEGs were retrieved from Ensembl Plants (http://plants.ensembl.org/index.html). Mapping of these genes was achieved using MapChart software [59].

### 4.6. Quantitative real-time PCR verification

The total RNA extraction, cDNA synthesis and qRT-PCR analysis were performed as described previously [60]. All primers applied in qRT-PCR were listed in electronic supplementary material, table S11. The PCR thermal profile was under the following cycle conditions: an initial 95°C for 15 s, followed by 40 cycles at 95°C for 10 s, and 60°C for 31 s. All data were generated from averages of three independent replicates and the relative expression levels of each gene were calculated using 2^−ΔΔCT^ method [61].

### 4.7. Gene ontology and Kyoto Encyclopaedia of Genes and Genomes enrichment analyses

GO and KEGG enrichment analyses of genes were implemented in topGO [62] and KOBAS 3.0 [63], respectively.

### 4.8. Net photosynthetic rate measurement

Light intensity in the open field and in shaded conditions under chestnut trees of 10 and 15 years old was measured on a sunny day at four time points (9.00, 11.00, 15.00 and 17.00), and mean values of the four time points for each condition were calculated and considered as the light intensity in open field and shaded conditions under chestnut trees of 10 and 15 years old. The first, second and third leaves from the top of each plant were sampled from 22 varieties of foxtail millet at booting stage, and net photosynthetic rates were measured by using a portable gas exchange fluorescence system (GFS-3000, WALS), with the light intensity set to the three mean values determined in the open field and shaded conditions under chestnut trees of 10 and 15 years old, respectively. The average value of net photosynthetic rates, which were obtained from the first, second and third leaves from the top of plants, were considered as the net photosynthetic rate of each variety in open field or shaded conditions under chestnut trees of 10 and 15 years old.

### 4.9. Weighted gene co-expression network analysis and module identification

ImageGP online analysis tool (http:/www.ehbio.com/Cloud_Platform/front/#/) was used for WGCNA. Scale-free co-expression network analysis was performed based on log_2_(FPKM + 1) of expressed genes (FPKM > 1). To satisfy the precondition of scale-free network distribution, the soft thresholding power of *β* was calculated by choosing the smallest one. In this study, we set the value of *β* to 28 (*R*^2^ = 0.85), as determined by assessment of scale-free topology (electronic supplementary material, figure S1). The modules grouped by WGCNA were then related to the ratio of net photosynthetic rate under shaded conditions to that in light conditions, and the corresponding module genes were candidates for subsequent analysis.

## Data Availability

All sequence data have been submitted to Sequence Read Archive (SRA) of the National Center for Biotechnology Information (NCBI) under the accession number PRJNA772942. The data are provided in the electronic supplementary material [64].
